# Identification of the Major Expressed S-Layer and Cell Surface-Layer-Related Proteins in the Model Methanogenic Archaea: *Methanosarcina barkeri* Fusaro and *Methanosarcina acetivorans* C2A

**DOI:** 10.1155/2012/873589

**Published:** 2012-05-15

**Authors:** Lars Rohlin, Deborah R. Leon, Unmi Kim, Joseph A. Loo, Rachel R. Ogorzalek Loo, Robert P. Gunsalus

**Affiliations:** ^1^Department of Microbiology, Immunology, and Molecular Genetics, University of California, Los Angeles, CA 90095, USA; ^2^UCLA-DOE Institute for Genomics and Proteomics, University of California, Los Angeles, CA 90095, USA; ^3^Department of Chemistry and Biochemistry, University of California, Los Angeles, CA 90095, USA; ^4^Mass Spectrometry Resource, Boston University School of Medicine, 670 Albany Street, Rm 511, Boston, MA 02118, USA; ^5^OPX Biotechnologies, Inc. Research Division, 2425 55th Street, Boulder, CO 80301, USA; ^6^Department of Biological Chemistry, University of California, Los Angeles, CA 90095, USA

## Abstract

Many archaeal cell envelopes contain a protein coat or sheath composed of one or more surface exposed proteins. These surface layer (S-layer) proteins contribute structural integrity and protect the lipid membrane from environmental challenges. To explore the species diversity of these layers in the Methanosarcinaceae, the major S-layer protein in *Methanosarcina barkeri* strain Fusaro was identified using proteomics. The Mbar_A1758 gene product was present in multiple forms with apparent sizes of 130, 120, and 100 kDa, consistent with post-translational modifications including signal peptide excision and protein glycosylation. A protein with features related to the surface layer proteins found in *Methanosarcina acetivorans* C2A and *Methanosarcina mazei* Goel was identified in the *M. barkeri* genome. These data reveal a distinct conserved protein signature with features and implied cell surface architecture in the Methanosarcinaceae that is absent in other archaea. Paralogous gene expression patterns in two *Methanosarcina* species revealed abundant expression of a single S-layer paralog in each strain. Respective promoter elements were identified and shown to be conserved in mRNA coding and upstream untranslated regions. Prior *M. acetivorans* genome annotations assigned S-layer or surface layer associated roles of eighty genes: however, of 68 examined none was significantly expressed relative to the experimentally determined S-layer gene.

## 1. Introduction

Like cell envelopes of other archaeal species as well as gram-positive and gram-negative bacteria, the envelopes of methanogenic archaea have essential roles in protecting the cell from environmental challenges [1–3]. For example, envelopes resist attacks directed at the cytoplasmic membrane by extracellular enzymes, small lipophilic or chaotrophic molecules, and other toxic agents. The envelopes also aid in resisting osmotic stress and dehydration while allowing transit of small molecular weight nutrients and waste products [4]. However, relatively little is known about the cell envelopes of the Methanosarcinaceae, which include highly studied model organisms *Methanosarcina acetivorans* C2A, *Methanosarcina mazei* Goe1, and *Methanosarcina barkeri* Fusaro. Prior electron microscopy studies reveal the presence of a typical S-layer surrounding the cytoplasmic membrane [5, 6]. Bioinformatic studies have predicted surface-layer and surface-layer-related proteins for these methanogenic strains. For example, the genome annotations of *M. acetivorans* list 81 ORFs with these assigned functions [7], while over 14 and 52 ORFs were annotated in the *M. mazei,* and *M. barkeri* genomes to code related surface layer proteins, respectively [8, 9]. In another study using a comparative bioinformatics approach, *M. mazei, M. barkeri*, and *M. acetivorans* were predicted to possess 12, 12, and 3 putative S-layer proteins, respectively [10]. There was little overlap of these gene predictions with the above annotations.

Little data exist that experimentally addresse the above predictions except for recent proteomic reports that identified major surface layer proteins in two strains, *M. mazei* Goe1 and *M. acetivorans* C2A [11]. The *Methanosarcina* studies revealed a protein in each species with a similar predicted amino acid sequence (i.e., MM1976 and MA0829), but differing in apparent size as revealed by SDS-PAGE. The *M. mazei* S-layer displayed three species of approximately 131, 119, and 101 kDa in size, each possessing glycan modifications of unknown composition. *M. acetivorans* displayed major S-layer protein forms 134, 119, and 114 kDa in apparent size [11]. Interestingly, these proteins were previously annotated as hypothetical proteins in the *M. mazei* and *M. acetivorans* genomes in contrast to the numerous other proteins annotated as surface layer or surface-related [7–9]. Based on protein homology searches to MM1976 and MA0829, the *M. mazei* and *M. acetivorans* genomes contained four to seven related ORFs [11]. The roles and expression of these related ORFs plus those previously annotated as surface associated in these model *Methanosarcina* strains remain unclear.

To address the above questions, combined proteomic, bioinformatic, and gene expression studies were performed to explore the diversity of surface layers in two model *Methanosarcina* strains. The major S-layer protein in *M. barkeri* was identified (Mbar_A1758) and its sequence was used to define a family of paralogous and orthologous proteins in the *Methanosarcinaceae*. Transcript levels of abundantly expressed ORFs from two model strains were examined and paralogous genes were identified. Finally, the expression of many *M. acetivorans* genes previously annotated as S-layer and surface-layer-associated proteins was examined: none were found to be significantly expressed. Together, these *M. acetivorans, M. mazei*, and *M. barkeri* studies reveal the presence of a distinct family of S-layer genes/proteins that support a bioinformatics-based reassessment of *Methanosarcinaceae *cell surface layers.

## 2. Methods and Materials

### 2.1. Cell Culture

*M. acetivorans* C2A (DSM 2834) and *M. barkeri *Fusaro (DSM 804) were cultivated on a mineral salts-based medium in their single cell forms as described previously [12] with an atmosphere (80 : 20) of nitrogen and carbon dioxide in the vessel headspace. Following sterilization, the medium was supplemented with filter-sterilized 0.1 mL 50% methanol or 0.2 mL 5 M acetate per 10 mL medium as previously described for *M. acetivorans* [13]. For *M. barkeri* cell growth, cultures were grown either with methanol (0.5% v/v) or with an 80 : 20 atmosphere of hydrogen : carbon dioxide in the vessel headspace.

### 2.2. RNA Purification

 For RNA isolations, cultures of *M. acetivorans* or *M. barkeri* were grown on the indicated substrates with serial transfer for a minimum of three times to midexponential phase prior to cell harvest. Total RNA was purified from 10 mL of cell samples using the RNAwiz (Ambion Austin, TX) following the manufacturer's instructions and as described [12]. The purified RNA was treated with DNase I as described [14, 15] and stored at −70°C until used.

### 2.3. Quantitative RT-PCR

Real-time reverse transcription (RT-PCR) reactions were performed using Superscript II (Invitrogen) as previously described [12]. To remove complementary RNA, 1 *μ*L RNase H was added to mixture and incubated for 20 min at 37°C. The real-time PCR reactions were conducted on a Biorad iCycler (Biorad, Hercules, CA) using a four-step program consisting of denaturing, annealing, extension, and acquisition steps. The RT-PCR primers were created by a modified version of MyPROBES [15]. The PCR product lengths were 100–200 bp where the melting temperature was in the range of 55–66°C. The GC content was 55–65%, and the primer length was 17–22 bases (see the supplementary material available online at doi:10.1155/2012/873589, Table  S1). Each primer pair was calibrated using genomic DNA [12]. Gene expression values were determined in triplicate and compared to a reference gene that showed no significant variation in expression in the *M. acetivorans *microarray experiments (i.e., MA3998), or with genomic DNA for the *M. barkeri *experiments. Experiments were performed in triplicate, and the standard deviations were less than 5%. Error bars are indicated in the appropriate figures. Abundance units (AUs) are expressed in copy number per 5 ng RNA [12].

### 2.4. Primer Extension Analysis

 Primer extension reactions were performed to determine the mRNA 5′ ends using-gene specific primers which were located 60 bases downstream of the ATG start codon of the *slmA*1^mba^ gene (Mbar_A1758) and 100 bases for *slmA*1^mac^ (MA0829) (Table  S1 listing each primer). Total RNA was isolated as described above. A total of 30 *μ*g of RNA was used in each primer extension reaction and carried out as describe in [12], but the Sequitherm Excel II Kit (Epicentre Madison, WI) was used to create ladder from sequencing reactions of unrelated DNA.

### 2.5. SDS-Polyacrylamide Gel Electrophoresis and Protein Visualization

 Proteins were resolved on NuPAGE 4–12% Bis-Tris gels using MES Running Buffer (Invitrogen) as previously described [11]. Images of stained proteins using SYPRO Ruby (Bio-Rad) were captured using a Molecular Imager FX scanner and PDQuest Image Analysis Software (Bio-Rad). Glycosylated proteins were revealed by Pro-Q Emerald 300 Glycoprotein Stain following the manufacturer's protocol (Molecular Probes) as previously described [11]. Protein molecular weight standards were obtained from Molecular Probes (Candy Cane glycoprotein standards) and Bio-Rad (biotinylated broad range protein standards).

### 2.6. Trypsin Proteolysis and Nano-HPLC-MS/MS

Protein bands to be identified were excised from SDS-PAGE gels by a spot-excision robot (Proteome Works, Bio-Rad) or by hand. The gel-embedded proteins were reduced, iodoacetamide-alkylated, and trypsin-digested (Promega, sequencing grade modified trypsin). Product peptides were extracted from the polyacrylamide matrix in 50% acetonitrile/0.1% trifluoroacetic acid in water and dried by vacuum centrifugation. Peptides were dissolved in 10 *μ*L of 0.1% formic acid (FA) solution and analyzed by liquid chromatography-tandem mass spectrometry (LC-MS/MS) with electrospray ionization (ESI) on an Applied BioSystems QSTAR Pulsar XL (QqTOF) mass spectrometer as previously described [11]. Tandem mass spectra were recorded automatically during the liquid chromatography run by information-dependent analysis (IDA) on the mass spectrometer with collision energies selected by the software to yield maximum fragmentation efficiency [11]. Database searches were performed on the MS/MS data utilizing Mascot (Matrix Science) and the complete MSDB database. Protein sequence searches employed one missed cleavage and a mass tolerance of 0.3 Da for both precursor and product ions. Protein hits were accepted based on ≥2 ascribed peptides, at least one of which possessed a MOWSE score ≥ 50 (*P* ≤ 0.05). Correspondences between MS/MS spectra and ascribed sequences were also verified manually.

### 2.7. Informatics Analysis and Data Visualization

Protein similarities were determined using BLAST [16] whereas the alignment and the phylogenetic tree of proteins were performed with clustalw [17]. The visualization of the trees was performed with splitTree4 [18]. Upstream DNA regions were searched for palindromic and repeated motifs using simple Perl script software written in house as were searches for conserved elements in the UTR regions [12]. Potential Rho-independent terminator sequences were predicted using TransTermHP software [19]. The signal peptides were predicted using signalP 3.0 [20] and the transmembrane domains were predicted using TMHMM [21]. The RNA secondary structures were predicted using RNAfold webserver [22].

## 3. Results

### 3.1. Identification of the Major *M. barkeri* Cell Surface Protein

**Figure 1:**
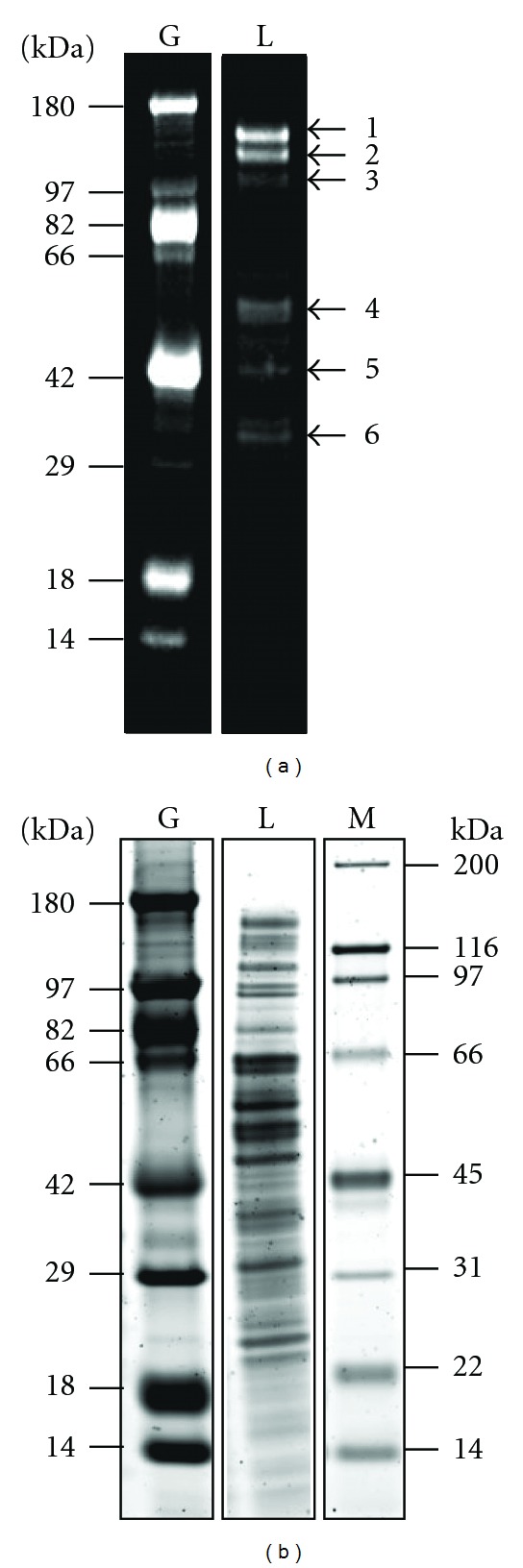
Fractionation of *M. barkeri* cell proteins by SDS-PAGE. (a) Pro-Q Emerald glycoprotein-stained gel. Lanes: G: glycoprotein standards; L: whole-cell lysate. Gel bands analyzed by LC-MS/MS are numbered. (b) SYPRO-Ruby-stained gel. Lanes: G: glycoprotein standards; L: whole-cell lysate; M: protein standard set 2. Protein sizes are indicated in kDa. The indicated bands 1–6 were excised for LC-MS/MS analysis.

We recently identified the major S-layer proteins in *M. acetivorans* C2A and *M. mazei* Goe1 to be the MA0829 and MM1976 gene products, respectively [11]. However, the identity and size(s) of the major *M. barkeri *S-layer protein(s) are currently unknown due to the presence of multiple paralogous genes related to the secreted and modified major surface proteins of *M. acetivorans* and *M. mazei* (described below). To address these questions, a proteomic analysis was performed using *M. barkeri *cells grown to midexponential phase with methanol as the sole source of carbon and energy (Section 2). Cell-extracted proteins were separated by SDS-PAGE and were visualized by fluorescence staining, employing SYPRO Ruby to reveal the total protein content (Figure 1(b)) and Pro-Q Emerald (Figure 1(a)) to reveal glycosylated proteins. Three prominent glycosylated species with apparent sizes of 130, 120, and 100 kDa were revealed by the Pro-Q Emerald stain (Figure 1(a), bands 1–3). The 130 kDa species appeared predominant by the SYPRO Ruby total protein stain (Figure 1(b)). In addition, several bands smaller in size also contained glycoproteins (Figure 1(a), marked as bands 4–6).

The major glycan-stained *M. barkeri *protein bands (Figure 1(a), marked 1–3) were excised from the SDS-PAGE gels and subjected to LC-MS/MS analysis (Table  S2). Highly abundant in each band was the protein encoded by Mbar_A1758. This gene is predicted to encode a hypothetical protein of 73,535 Da in size ([9] IMG JGI) but because the gel exhibited species with 130, 120, and 100 kDa, it appears that the protein is posttranslationally modified, as are the S-layer proteins of *M. acetivorans* and *M. mazei* ([11]; discussed below). By tandem mass spectrometry, the mature N-terminus (ADSVEIR) was determined to begin with residue 25 (Table  S3). 

### 3.2. The *M. barkeri* Genome Possesses Nine Mbar_A1758-Like Proteins

 A bioinformatic search for Mbar_A1758-like proteins encoded in the *M. barkeri *Fusaro genome (Section 2) revealed nine highly conserved ORFs (Table 1). All paralogs are predicted to possess signal peptides that suggest protein export, plus one or two Pfam domains of unknown function called DUF1608. However, the proteins vary in apparent size and in the presence of a C-terminal hydrophobic transmembrane element.

**Figure 2:**
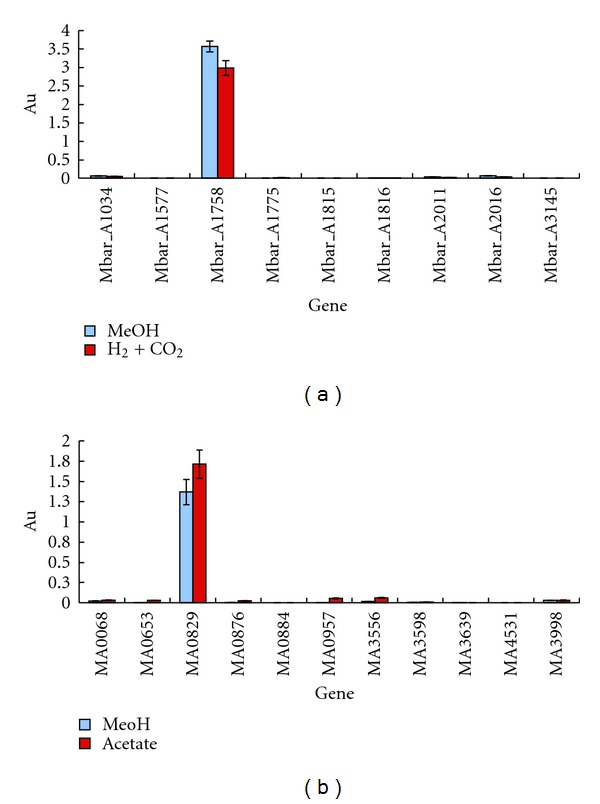
Expression of *M. acetivorans* and *M. barkeri* paralogous S-layer genes. (a) The Mbar_A1758-related genes in *M. barkeri.* (b) The MA0829-related genes in *M. acetivorans.* Abundance values (AU) are expressed in copy number [12]. Cells were grown with methanol (MeOH), acetate, or hydrogen and carbon dioxide (H_2_ + CO_2_) as the sole carbon and energy supply as described in Section 2.

To determine which paralogs were abundantly expressed in *M. barkeri*, unique primer pairs were designed for each gene and employed in a quantitative PCR-based gene transcription assay (Figure 2(a); Section 2): RNA was isolated from cells grown to mid-exponential phase with either methanol or hydrogen/carbon dioxide as sole source of carbon and energy. Only one of the *M. barkeri* ORFs (i.e., Mbar_A1758) was abundantly expressed. Of the remaining eight genes, transcripts for only two, Mba_A1034 and Mbar_A2016, were detected (*ca*., at 1.7 and 1.2% of the Mbar_A1758 transcripts). Based on abundant detection of the Mbar_A1758 protein in *M. barkeri* cell extracts and in the uniquely high level of its transcript, it appears to constitute the major envelope S-layer protein. For subsequent gene identification and description, we designate this protein/gene as *SlmA*1^mba^/*slmA*1^mba^.

### 3.3. The *M. acetivorans* Genome Possesses Multiple MA0829-Like Genes

A prior Pandit phylogeny search of the *M. acetivorans *genome [11] revealed four proteins related to MA0829 (i.e., MA0068, MA0844, MA3556, and MA3639). All of these were predicted to encode proteins that contained domains of unknown function, designated as DUF1608. In an expanded bioinformatic search of the *M. acetivorans *genome (Section 2) six additional ORFs were identified of varying sizes from 34 to 131 kDa (Table 1). Interestingly, all have been annotated as hypothetical proteins of unknown function ([7]; discussed below) and possess one or two DUF1608 domains.

### 3.4. The *M. acetivorans* MA0829 Gene is Abundantly Expressed

 To determine which of these ten MA0829-like genes in *M. acetivorans* genome were expressed, unique DNA primer pairs were designed for each as described above for* M. barkeri* (Section 2, Table  S1) and cells were grown to mid-exponential phase with either methanol or acetate as the sole carbon and energy supply. Total cellular RNA was isolated and employed in a quantitative PCR-based gene expression assay (Figure 2(b)). With the one exception (ORF MA0829, designated *slmA*1^mac^ to distinguish it from the related ORFs) all transcripts were present in low to nearly undetectable abundance. Relative to *slmA*1^mac^, the next most highly expressed ORF was MA3556 (*slmA*2^mac^) followed by MA0068 (*slmA*3^mac^) at levels of approximately 3.5 and 1.8%, respectively. Interestingly, our prior proteomic study, which enriched for surface proteins, detected the MA0068 and MA3556 proteins from Concanavalin A eluates, suggesting that the proteins are either glycosylated or interact with glycans. They were not detected without employing Concanavalin A enrichment [11]. Potential roles of these paralogous *slmA1*-like genes are discussed below.

### 3.5. Identification of the MA0829 mRNA 5′ End

**Figure 3:**
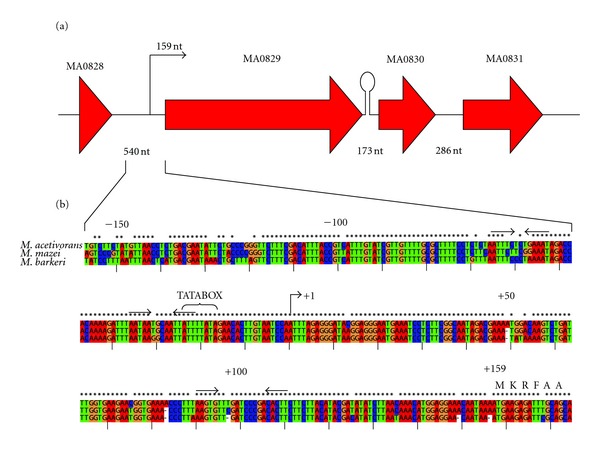
The *M. acetivorans* MA0829 gene locus and mRNA 5′ end. (a) The MA0829-MA0831 gene region encodes the S-layer protein (MA0829), plus two hypothetical proteins of unknown function (MA0830 and MA0831). The Genebank name (MA number) is shown above each gene. A predicted hairpin loop is indicated between MA0829 and MA0830. Intergenic distances are in nucleotides. (b) Alignment of the upstream and untranslated leader regions of genes encoding the *Methanosarcina* S-layer protein. The alignment of the upstream DNA sequences is relative to the start of transcription (+1 position for *M. acetivorans slmA1,* see text) while the ATG position of the initiation codon is indicated by the M. Numbering is relative to the transcription start. Identity of the sequences is indicated by asterisks. The putative TATA-box sequences are indicated by the bracket. The mRNA 5′ end positions for *M. acetivorans* and *M. barkeri* were determined with a ubiquitous ladder (data not shown). The six first N-terminal amino acids for the unprocessed *M. acetivorans* MA0829 preprotein are indicated by the single amino acid code.

 The *M. acetivorans slmA1 *transcript's 5′ end was determined (Section 2). A single 5′ terminus was observed corresponding to a position located 159 nucleotides upstream of the *slmA1* translational start (Figure 3(a), bent arrow). Analysis of the DNA coding region upstream of the mRNA initiation site contained a recognizable TATA box needed for TBP recognition/recruitment and DNA binding [12]. Three short regions of dyad symmetry of 14 to 21 nucleotides in length were also seen in this region (Figure 3(b), thin arrows). Finally, inspection of the 5′ mRNA untranslated region (UTR) revealed several potential RNA secondary structures (Section 2: Figure  S1). 

**Figure 4:**
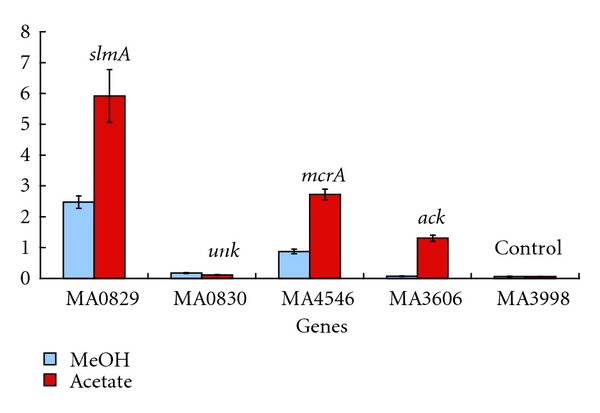
Expression levels of the MA0829 and MA0830 genes in *M. acetivorans* relative to methyl-Coenzyme M transferase (*mcrA*) and acetate kinase (*ack*) genes. RT-PCR expression data for the indicated genes in cells grown on methanol versus acetate as the carbon supply. Abundance values (AU) are expressed as copy number [12].

The *M. acetivorans slmA1* gene is located upstream of two genes that encode hypothetical proteins (i.e., MA0830-MA0831) and downstream of a tRNA gene (Figure 3). To determine if the MA0830 ORF is cotranscribed with *slmA1*, quantitative PCR (qPCR) experiments were performed (Figure 4). MA0830 expression was about 2% (acetate) to 7% (methanol) of the level seen for *slmA1*, suggesting that little to no transcription read-through occurs from the upstream MA0829 promoter element. There is a rho-independent-like terminator sequence after MA0829 predicted using TransTermHP [19]. The proteomic studies provided no evidence for significant amounts of either MA0830 or MA0831.

To compare *slmA1* gene expression levels relative to several highly abundant ORFs that function in *M. acetivorans* methanogenesis, specific primer pairs (Table  S1) were designed for the *mcrA* and* pta* genes. Among the most highly expressed in the cell, the *mcrA* gene encodes the A subunit of the methyl coenzyme M reductase enzyme of the central pathway in methane formation, while *pta *encodes phosphotransacetylase required for acetate activation and utilization. The resulting qPCR analysis demonstrated that *slmA1* expression was significantly higher (*ca*. by 2 to 10-fold) than for either *mcrA* or* pta* (Figure 4).

The *M. barkeri slmA1* mRNA 5′ end was also identified (Section 2, Figure 3(b)) and it corresponds to a position analogous to that found for *M. acetivorans slmA1*. Located 154 nucleotides (nt) upstream of the translational start site, *M. barkeri slmA1* has a UTR sequence highly conserved in relation to that of *M. acetivorans *(*>*91% identity). Further upstream, the DNA also contained a conserved TATA box. Interestingly, an annotated hypothetical protein coded directly downstream of *slmA1, *Mbar_A1759, was identified in this proteomic study (data not shown). However, that gene was only weakly transcribed in methanol grown cells relative to Mbar_A1758, and it does not appear to be significantly accumulated by *M. barkeri*.

### 3.6. The *M. mazei* Genome Possesses Five Mbar_A1758-Like Proteins

A search of the *M. mazei* Goe1 genome for *M. acetivorans *MA0829-like and *M. barkeri* Mbar_A1758-like proteins (Section 2) revealed five highly conserved ORFs (Table 1, described below). One of these, MM1976 (*slmA*1^mm^), was previously identified to be the major S-layer protein on the *M. mazei* cell surface [11].

### 3.7. Are the Previously Annotated *M. acetivorans* S-Layer and Surface-Related Proteins Abundantly Expressed?

The annotation of the *M. acetivorans *genome lists approximately eighty ORFs with assigned functions as S-layer proteins or surface-related proteins [7] (Table  S4). To determine if any of these ORFs were significantly expressed, we analyzed two *M. acetivorans *microarray data sets from cells grown under the conditions with acetate or with methanol as the sole supply of carbon and energy (Section 2). The compiled pixel data sets were then normalized to the *mcrA* gene that encodes methyl coenzyme M reductase (MA4546). Strikingly, none of the sixty eight previously annotated ORFs for which we had data were significantly expressed relative to *mcrA* (*ca.*, below 3-4%). As noted above, *slmA*1^mac^ (MA0829) expression was 2–4-fold above that observed for the *mcrA* gene (Figure 4). Lastly, the twelve surface layer proteins predicted for *M. acetivorans* by Saleh et al. [10] constituted a subset of those shown in Table  S3: they were not significantly expressed relative to MA0829. These experimental findings indicate limitations of the computational tools used to predict archaeal S-layer proteins and surface associated proteins for this group of methanogens. 

## 4. Discussion

### 4.1. Identification and Properties of the *M. barkeri* Surface Layer Protein

Based on the prior *M. acetivorans *and* M. mazei *S-layer proteomic and bioinformatic data, it was not possible to predict which of the three to five closely related ORFs in the *M. barkeri* genome encoded the major S-layer protein(s) of this microbe. Glycoprotein staining of SDS-PAGE separated *M. barkeri* cell extracts revealed three distinct bands containing the Mbar_A1758 polypeptide (see Pro-Q Emerald glycoprotein stained lanes 1–3, Figure 1(a)). This *M. barkeri *protein is analogous to the surface exposed *M. acetivorans* and *M. mazei* S-layer proteins revealed by biotin-tagging studies [11]. It is posttranslationally processed by removal of the N-terminal signal sequence and further modified by addition of unknown sugar moieties. The mature N-terminus (ADSVEIR) was determined to begin with residue 25 (Table  S4) and corresponds to those determined for MA0829 and MM1976 [11]. Lectin blotting revealed interactions between Mbar_A1758 and the lectins Con A, *Galanthus nivalis* lectin (GNL), and *Pisum sativum* agglutinin (PSA). Con A, preferentially binding *α*-mannose and *α*-glucose, has previously been shown by us to bind the *M. mazei* and *M. acetivorans* S-layer proteins. GNL binds (*α*-1,3) mannose residues preferentially and, unlike most mannose-binding lectins, does not bind *α*-glucose. PSA prefers to bind *α*-mannose-containing oligosaccharides with *N*-acetylchitobiose-linked *α*-fucose residues. These lectin results suggest future approaches to enrich Mbar_A1758 from whole-cell lysates.

### 4.2. The Highly Expressed *Methanasarcina slmA1* Genes

 The *M. acetivorans *MA0829 gene (*slmA*1^mac^) was among the most highly expressed in the cell (Figures 2 and 4). Interestingly, the downstream gene, MA0830, was not significantly transcribed and does not appear to be part of an operon-like structure. The *M. barkeri slmA1* gene (Mbar_A1758) was also highly transcribed from an identical upstream transcription start site (Figure 3(b)). The high conservation among the UTRs and promoter elements for the predominant *slmA1* genes from *M. acetivorans, M. mazei*, and* M. barkeri *suggests that all three organisms control transcription and translation of their S-layer proteins in a similar way. The role of the UTR and putative secondary mRNA structures (Figure S1) in gene expression is currently unknown.

While the Mbar_A1758 and MA0829 proteins are the most abundantly expressed S-layer proteins in *M. barkeri *and *M. acetivorans*, two additional proteins, MA0068 and MA3556, were detected by proteomic methods, albeit at much lower levels [11]. Correspondingly, their genes were transcribed at far lower levels relative to MA0829 (i.e., at less than 0.04 and 0.02 of the level, resp., Figure 2(b)). Whether they represent minor components of the S-layer somehow needed for envelope porosity and/or proper assembly is unknown. Alternatively, they may be more highly expressed under different cell growth conditions. Likewise, for the Mbar_A1034 and Mbar_A2016 gene products detected in *M. barkeri.*

Alignment of the primary amino acid sequences of the three major S-layer proteins from *M. barkeri*, *M. acetivorans*, and *M. mazei *(Figure 6) reveals high conservation (65% identity and 88% similarity). Each protein has a similar signal peptide sequence targeting secretion followed by tandem DUF1608 domains and a short 60 amino acid linker region. The latter element would presumably tether the S-layer protein to the cytoplasmic membrane by a C-terminal and hydrophobic transmembrane helix. A more detailed assessment of these proteins and their respective paralogs is in progress.

### 4.3. Mbar_A1758-Like Proteins in Other Microorganisms

**Figure 5:**
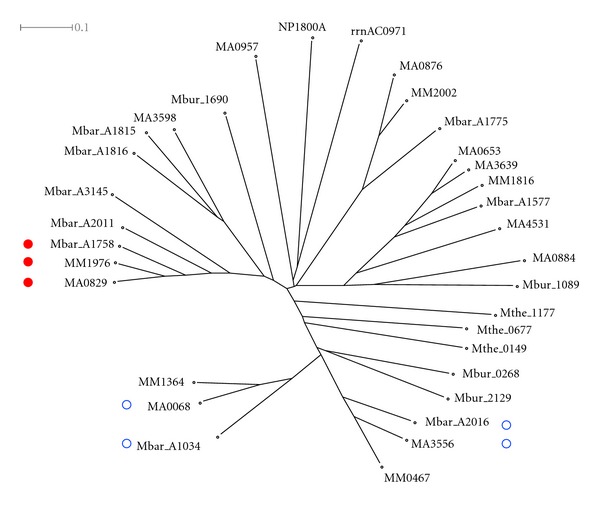
Phylogenetic tree of all DUF1608-containing genes in sequenced genomes of *Methanosarcina *and in other archaeal species. The organisms are: *M. acetivorans*, MA; *M. mazei*, MM; *M. barkeri*, Mbar_A; *M. burtonii*, Mbur_; *Methanosaeta thermophila* PT., Mthe_; *Haloarcula marismortui*, rrnAC; *Natronomonas pharaonis* DSM2160, NP. The indicated major (•) and minor (°) expressed S-layer proteins are described in the text.

**Figure 6:**
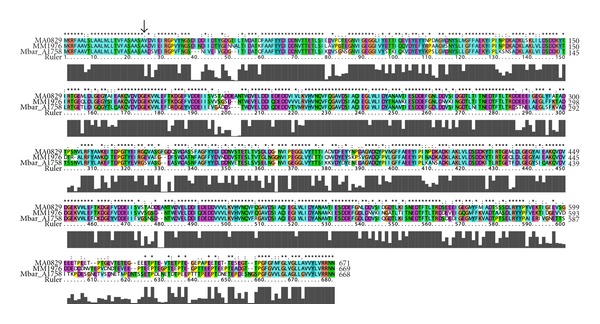
Primary amino acid alignment of the three major *Methanosarcina* S-layer proteins, Mbar_A1758, MA0829, and MM1976. Identity of the sequences is indicated by asterisks. The experimentally determined mature N-terminus for Mbar_A1758 (Section 3) is indicated by the arrow.

Mbar_A1758-like proteins identified in the sequenced genomes of *Methanosarcina* species and other microorganisms (Section 2, Table 1) are displayed in the phylogenetic tree shown in Figure 5. Four major conclusions may be drawn. First, these proteins group into six deeply branched clusters with the *M. acetivorans* MA0829 (*slmA*1^mac^) and the *M. mazei *MM1976 (*slmA*1^mm^) proteins next to one another, and adjacent to three *M. barkeri* ORFs (Mbar_A1758, Mbar_A2011, and Mbar_A3145). An additional two *M. barkeri* ORFs (Mbar_A1815, Mbar_A1816) are the next most closely related proteins. Thus, it was not possible to predict in advance which of these five *M. barkeri *genes might encode the major S-layer protein for this organism. From the gene expression studies (Figure 3(b)), Mbar_A1758 is the logical candidate due to its high level of transcription relative to all of the other related genes. Moreover, proteomic studies show that the Mbar_A1758 gene product is highly abundant, proteolytically processed, and further modified by unknown glycan additions.

 Second, while the number of MA0829 homologs in *M. acetivorans* and *M. barkeri* is similar (i.e., ten and nine proteins, resp.), *M. mazei *only possesses five (Table 1). Interestingly, all of these appear to be closely related to an *M. acetivorans* and *M. barkeri* ORF (Figure 5), where only one *Methanosarcina* cluster lacks a *M. mazei *member. Four of the five *M. mazei *ORFs have been detected by their binding to Con A (Leon et al., in preparation) and only MM2002 has not been observed. *M. acetivorans* proteins MA3556 and MA0068 were readily detected from Con A elutes, while proteins MA0653 and MA4531 were observed less consistently. Noteworthy, the major ORFs detected in our *Methanosarcina* studies fall into three distinct groups on the phylogenetic tree (Figure 5). One contains the most abundantly detected proteins while the other two are less abundant.

 Third, only two other methanogenic archaea possess MA0829-like genes/proteins. These are *Methanococcoides burtonii* (four homologs: Mbur_0268, Mbur_1089, Mbur_1690, and Mbur_2129) and *Methanosaeta thermophila* PT (three homologs: Mthe_0148, Mthe_0677, and Mthe_1177). None of these putative proteins appear to be closely related to any of the abundantly identified *Methanosarcina* S-layer ORFs, but rather cluster together near the more distant and weakly expressed *Methanosarcina* genes (Figure 5). Interestingly, the Mbur_1690 ORF was detected in the cell-free supernatant of *M. burtonii *cultures [23], and this protein is most similar to the S-layer proteins detected in *M. acetivorans* and *M. mazei* [11].

 Fourth, only two nonmethanogenic archaea possess MA0829-like genes/proteins (Figure 5). These include one each in *Haloarcula marismortui* (rrnAC0971, UniProt Q5V3G1) and in *Natronomonas pharaonis* DSM2160 (NP1800A, UniProt Q3IS97). No related ORFs were identified in either the Bacteria or the Eukarya. The MA0829 protein appears to be relatively rare in a phylogenetic context, but at the same time, it is one of the major proteins in these few organisms known to possess it.

### 4.4. Previously Annotated Surface Layer and Surface-Layer-Associated Proteins

 While multiple S-layer and surface-layer-associated genes were annotated by the *Methanosarcina* genome sequencing projects [7–9], it is now evident that experimental data were too limited to accurately predict the S-layer proteins present in these archaeal species. Interestingly, the bioinformatic study of Saleh et al. [10] predicted yet different S-layer proteins with little overlap to other predictions. However, an analysis of several microarray data sets for *M. acetivorans* failed to support the abundant expression of the above-mentioned candidates (Table S4).

In conclusion, this study establishes the presence of a single major S-layer protein in each *Methanosarcina *strain examined. Its signature is well conserved within the Methanosarcinaceae but nearly absent in any other types of organisms. The current study revises criteria for predictions of newly sequenced genomes and metagenomic data sets. In fact, it will be interesting should future studies assign newly revealed *slmA1* genes/proteins to Methanosarcinaceae only. If so, the DUF1608 motif is restricted to a relatively narrow range of species.

## Supplementary Material

A list the oligonucleotides used in this study is given (supplementary Table 1). The list of proteins identified by LC-MS/MS is given (supplementary Table 2) as are the peptides of Mbar_A17S8 detected by LC-MS/MS (supplementary Table 3). The abundances of transcripts from 68 genes annotated as cell surface or surface-related in the M. acetivorans genome are given (supplementary Table 4). A proposed RNA secondary structure for the UTR of Mbar A17S8 is given (supplementary Figure 1).

## Figures and Tables

**Table 1:** Summary of the DUF1608-containg proteins in the methanogenic archaea.

ORF^a^	SignalP^b^	TM^b^	DUF1608 domains^b^	Mass kDa^a^	AA^a^
MA0068	+	1	2	127.0	1167
MA0653	− (+)	0 (1)	2	74.2	760
MA0829	+	1	2	74.4	671
MA0884	+	0	2	80.2	717
MA0876	+	0	1	65.2	585
MA0957	+	0	2	131.3	1196
MA3556	+	1	2	94.7	868
MA3598	+	0	1	52.4	475
MA3639	+	0	2	83.2	744
MA4531	−(+)	1	1	34.2	317
Mbar_A1034	+	1	2	125.5	1161
Mbar_A1577	+	0	2	82.4	744
Mbar_A1758	+	1	2	73.5	668
Mbar_A1775	+	0	1	65.2	586
Mbar_A1815	+	0	2	68.5	617
Mbar_A1816	+	0	2	55.6	505
Mbar_A2011	+	0	2	74.3	681
Mbar_A2016	+	1	2	94.4	868
Mbar_A3145	+	1	3	121.4	1096
MM0467	+	0	2	94.6	868
MM1364	+	1	2	126.2	1164
MM1816	+	0	1	46.5	425
MM1976	+	1	2	74.1	669
MM2002	−	0	1	63.2	564
Mbur_0268	+	1	2	95.7	867
Mbur_1089	+	1	1	43.6	396
Mbur_1690	+	0	2	73.9	677
Mbur_2129	+	1	2	139.4	1304
Mthe_0149	+	1	2	118.9	1113
Mthe_0677	+	0	2	79.9	720
Mthe_1177	+	1	2	96.8	874

^a^Gene and protein properties are derived from the original genome annotation files [7–9]. ^b^The signalP (SP), transmembrane (TM), and DUF1608 domains were predicted as noted in Section 2.
