# Supplementary material for: Identification of the Major Expressed S-Layer and Cell Surface-Layer-Related Proteins in the Model Methanogenic Archaea: Methanosarcina barkeri Fusaro and Methanosarcina acetivorans C2A
Source: Archaea. 2012 May 15;2012:873589. doi: 10.1155/2012/873589 (PMC3361143; doi:10.1155/2012/873589)
Supplement: Supplementary file 1 — A list the oligonucleotides used in this study is given (supplementary Table 1). The list of proteins identified by LC-MS/MS is given (supplementary Table 2) as are the peptides of Mbar_A17S8 detected by LC-MS/MS (supplementary Table 3). The abundances of transcripts from 68 genes annotated as cell surface or surface-related in the M. acetivorans genome are given (supplementary Table 4). A proposed RNA secondary structure for the UTR of Mbar A17S8 is given (supplementary Figure 1). [file 873589.f1.pdf]

Supplementary Materials

Figure S1: Potential RNA secondary structures located in the un-translated region (UTR) of the *M. barkeri* Mba_A1958 mRNA using RNAfold webserver [16]. Regions of potential base pairing are indicated as are the 5' mRNA start and the 3' end corresponding to the start of translation.

Table S1: Oligonucleotides used in this study.

Table S2: Proteins identified by LC-MS/MS.

Table S3: Mbar_A1758 peptides detected from analyses of multiple gel bands.

Table S4: Microarray transcript abundance of all cell surface and cell surface-related genes annotated in the *M. acetivorans* genome.

Figure S1: Potential RNA secondary structures located in the untranslated region (UTR) of the *M. barkeri* Mba_A1758 mRNA.

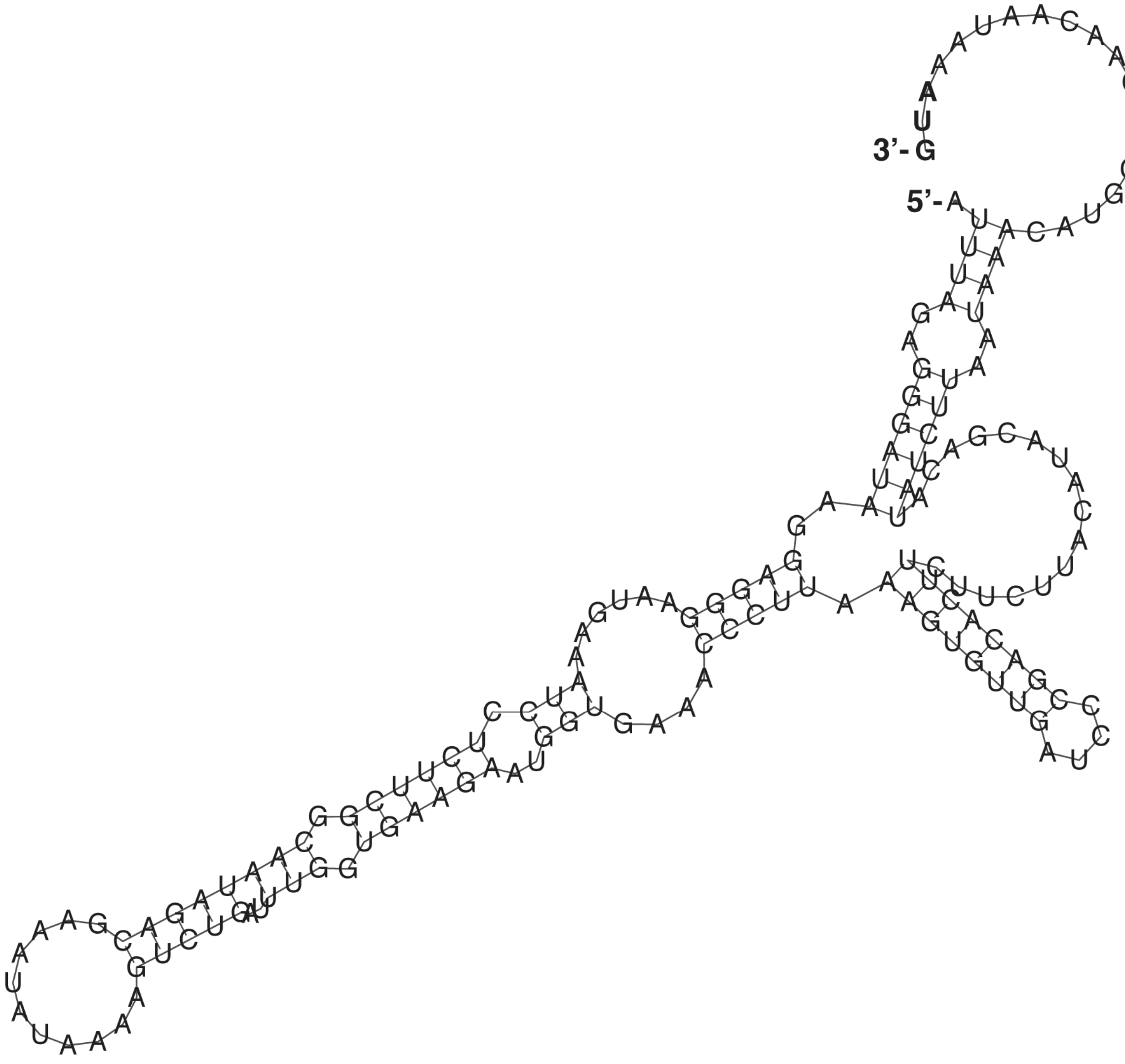

Table S1: Oligonucleotides used in this study.

Primer Name	Sequence	Primer Name	Sequence
Mbar_A1034RTfor	cactccaagttctgctcactcca	Mbar_A1034RTrev	tggcaggttgatggaagca
Mbar_A1577RTfor	catcctctgctcctcttctcga	Mbar_A1577RTrev	aactcctgcaagcacctcga
Mbar_A1758RTfor	gctgacactccaacatccga	Mbar_A1758RTrev	cggagtctcgctgctggta
Mbar_A1775RTfor	tcgttcggattcttctccaga	Mbar_A1775RTrev	ccagccgtcttcagcattaag
Mbar_A1815RTfor	tcaacctgcttggccttgatac	Mbar_A1815RTrev	actggttgccgattgcga
Mbar_A1816RTfor	tgccatggagtggataagctt	Mbar_A1816RTrev	agtcggagaattcacgcttga
Mbar_A2011RTfor	agcttgtcagcagcgttgg	Mbar_A2011RTrev	ggcaggactataggagataacggt
Mbar_A2016RTfor	ttccgatgattgcggtcc	Mbar_A2016RTrev	cattgtgatgcctgagtcgga
Mbar_A3145RTfor	tgcagagcttgctggcatc	Mbar_A3145RTrev	gtgtctctggagttggcgga
MA0068RTfor	gatgatgaggaggaggacactgt	MA0068RTrev	gccggacatcggatatgact
MA0653RTfor	cggctacgttattcatcctgca	MA0653RTrev	ttcctctgcggcttcttca
MA0829RTfor	cggcttcggtgatcaatca	MA0829RTrev	tgcgatggtggtggtgtaa
MA0876RTfor	Gaagatctctggatcggacggt	MA0876RTrev	ggacacggaagtggttgaagg
MA0884RTfor	Ggctccgaacatcctgca	MA0884RTrev	catctccgccgatagcca
MA0957RTfor	Cagaaccggtgtattcgctca	MA0957RTrev	tggataaccgtctgcgtgaag
MA3556RTfor	Tctgctcctcaaccttctgacc	MA3556RTrev	ttgctcctctcggtctctcc
MA3598RTfor	Gtgcgcctgttccgttga	MA3598RTrev	tgcatcgtcaggattgatgg
MA3639RTfor	Ccatgcatctccggtctacc	MA3639RTrev	tcttcagaggcgacaagatcct
MA4531RTfor	Tgaacattgctggaagcagg	MA4531RTrev	ggaagcaatggaggatacgtg
MA0830RTfor	Agccggaataagcacacttga	MA0830RTrev	ccttcggagacgttaagaacc
MA3606RTfor	Caatcgaaccgcacctgg	MA3606RTrev	gtcatcacctgccacctaggaa
MA4546RTfor	Ggcacatgcaacaccgga	MA4546RTrev	tcgcaaccgagtccacactc
MA3998RTfor	Caacacggtcgcagatctc	MA3998RTrev	ccaaggtggcggttacaa
Mbar_A1758prim	Cggatgcaaatacagtcagaagcatgaga gcagccag	MA08295prim	Ctggaccacggatctcaattacgtctactgcgctt

Table S2: Proteins identified by LC-MS/MS.

ID number (UniProt)	Comment	ID number (NCBI)	Protein Description	Mascot Prot Score	MW (Da)	Peptides
**Band 1**						
P0C0W7_METBF		Mbar_A1502	Trimethylamine methyltransferase mttB (EC 2.1.1.-) (TMA methyltransferase).	1140	53625	21
Q46BP2_METBF		Mbar_A1758	Hypothetical protein.	1097	73491	23
P07955_METBF		Mbar_A0897	Methyl-coenzyme M reductase subunit beta (EC 2.8.4.1) (Coenzyme-B sulfoethylthiotransferase beta).	836	45261	14
Q46CN6_METBF		Mbar_A1396	Pyruvate, phosphate dikinase.	706	96914	15
Q464Z4_METBF		Mbar_A3685	Protein translation elongation factor 1A.	672	46128	15
P07962_METBF		Mbar_A0893	Methyl-coenzyme M reductase subunit alpha (EC 2.8.4.1) (Coenzyme-B sulfoethylthiotransferase alpha).	560	61791	12
Q46D74_METBF		Mbar_A1201	Hsp60.	299	59181	6
Q464Z3_METBF		Mbar_A3686	Elongation factor 2 fusA EF2_METBF AAZ72549	299	80864	6
Q469Z7_METBF		Mbar_A2374	Carbamoyl-phosphate synthase large chain.	295	118270	8
Q46FD0_METBF		Mbar_A0199, Mbar_A0429	Carbon-monoxide dehydrogenase, subunit gamma.	220	51200	5
Q46CA1_METBF		Mbar_A1543	60 kDa chaperonin.	173	57890	4
Q46FC7_METBF or Q46G06_METBF		Mbar_A0432 or Mbar_A0202	Carbon-monoxide dehydrogenase, subunit beta.	169	52718	4
Q465G4_METBF		Mbar_A3613	Isoleucyl-tRNA synthetase..	169	120433	3
Q46DJ0_METBF		Mbar_A1084	Hsp60.	160	58194	4
Q46FV4_METBF		Mbar_A0254	F420-dependent N5,N10-methylene-tetrahydromethanopterin reductase.	150	34570	2
Q46EW5_METBF		Mbar_A0597	Phosphomethylpyrimidine synthase 1 thiC1 AAZ69577	133	46586	3
Q46FH4_METBF		Mbar_A0385	H(+)-transporting ATP synthase, subunit B.	108	50308	2
Q46E72_METBF, Q1DGD7_METBF, Q46E73_METBF, P0C0W3_METBF		Mbar_A0843 or Mbar_A0846	Monomethylamine methyltransferase mtmB1 (EC 2.1.1.-) (MMA methyltransferase 1) (MMAMT 1).. MTMB1_METBF=Q46E72	80	49945	2
**Band 2**						
Q46BP2_METBF		Mbar_A1758	Hypothetical protein.	966	73491	17
P0C0W7_METBF		Mbar_A1502	Trimethylamine methyltransferase mttB (EC 2.1.1.-) (TMA methyltransferase).	899	53625	16
Q46CN6_METBF		Mbar_A1396	Pyruvate, phosphate dikinase.	757	96914	15
Q464Z4_METBF		Mbar_A3685	Protein translation elongation factor 1A.	605	46128	14
P07955_METBF		Mbar_A0897	Methyl-coenzyme M reductase subunit beta (EC 2.8.4.1)	593	45261	11

ID number (UniProt)	Comment	ID number (NCBI)	Protein Description	Mascot Prot Score	MW (Da)	Peptides
			(Coenzyme-B sulfoethylthiotransferase beta).			
P07962_METBF		Mbar_A0893	Methyl-coenzyme M reductase subunit alpha (EC 2.8.4.1) (Coenzyme-B sulfoethylthiotransferase alpha).	492	61791	12
Q469Z7_METBF		Mbar_A2374	Carbamoyl-phosphate synthase large chain.	369	118270	9
Q46G04_METBF		Mbar_A0204	Carbon-monoxide dehydrogenase, subunit alpha.	256	88717	6
Q464Y7_METBF		Mbar_A3692	DNA-directed RNA polymerase, subunit A.	253	98377	9
Q465G4_METBF		Mbar_A3613	Isoleucyl-tRNA synthetase..	216	120433	5
Q464Z3_METBF		Mbar_A3686	Elongation factor 2 fusA EF2_METBF AAZ72549	197	80864	5
Q46AW0_METBF		Mbar_A2048	Leucyl-tRNA synthetase.	195	111025	6
Q46FC7_METBF or Q46G06_METBF		Mbar_A0432 or Mbar_A0202	Carbon-monoxide dehydrogenase, subunit beta.	193	52718	5
Q465I5_METBF		Mbar_A3589	Superoxide dismutase (Fe) (EC 1.15.1.1)..	182	23931	5
Q46AV5_METBF		Mbar_A2053	Membrane alanine aminopeptidase.	181	109644	5
Q46FD0_METBF		Mbar_A0199, Mbar_A0429	Carbon-monoxide dehydrogenase, subunit gamma.	156	51200	4
Q46CA1_METBF		Mbar_A1543	60 kDa chaperonin.	156	57890	3
Q46D74_METBF		Mbar_A1201	Hsp60.	153	59181	3
Q465Y6_METBF		Mbar_A3433	Heat shock 70 kDa protein; HSP70, chaperone dnaK_METBF AAZ72306:	135	66362	2
Q46C68_METBF		Mbar_A1577	Hypothetical protein.	132	82328	3
Q46E72_METBF, Q1DGD7_METBF, Q46E73_METBF, P0C0W3_METBF		Mbar_A0843 or Mbar_A0846	Monomethylamine methyltransferase mtmB1 (EC 2.1.1.-) (MMA methyltransferase 1) (MMAMT 1).. MTMB1_METBF=Q46E72	113	49945	4
Q46EW5_METBF		Mbar_A0597	Phosphomethylpyrimidine synthase 1 thiC1 AAZ69577	98	46586	2
**Band 3**						
Q46CN6_METBF		Mbar_A1396	Pyruvate, phosphate dikinase.	1498	96914	30
P0C0W7_METBF		Mbar_A1502	Trimethylamine methyltransferase mttB (EC 2.1.1.-) (TMA methyltransferase).	930	53625	17
Q464Y7_METBF		Mbar_A3692	DNA-directed RNA polymerase, subunit A.	890	98377	22
Q46BP2_METBF		Mbar_A1758	Hypothetical protein.	667	73491	12
Q464Z4_METBF		Mbar_A3685	Protein translation elongation factor 1A.	524	46128	12
P07962_METBF		Mbar_A0893	Methyl-coenzyme M reductase subunit alpha (EC 2.8.4.1) (Coenzyme-B sulfoethylthiotransferase alpha).	476	61791	10
P07955_METBF		Mbar_A0897	Methyl-coenzyme M reductase subunit beta (EC 2.8.4.1) (Coenzyme-B sulfoethylthiotransferase beta).	452	45261	8

ID number (UniProt)	Comment	ID number (NCBI)	Protein Description	Mascot Prot Score	MW (Da)	Peptides
Q464Z3_METBF		Mbar_A3686	Elongation factor 2 fusA EF2_METBF AAZ72549	402	80864	10
Q46FD0_METBF		Mbar_A0199, Mbar_A0429	Carbon-monoxide dehydrogenase, subunit gamma.	304	51200	6
Q46CS0_METBF		Mbar_A1361	Alanyl-tRNA synthetase.	265	103076	8
Q46G04_METBF		Mbar_A0204	Carbon-monoxide dehydrogenase, subunit alpha.	244	88717	6
Q465Y6_METBF		Mbar_A3433	Heat shock 70 kDa protein; HSP70, chaperone dnaK_METBF AAZ72306:	187	66362	3
Q46CA1_METBF		Mbar_A1543	60 kDa chaperonin.	162	57890	3
Q46FC7_METBF or Q46G06_METBF		Mbar_A0432 or Mbar_A0202	Carbon-monoxide dehydrogenase, subunit beta.	148	52718	4
Q46B32_METBF		Mbar_A1976	Valyl-tRNA synthetase valS SYV_METBF AAZ70910	129	98910	4
Q46D74_METBF		Mbar_A1201	Hsp60.	122	59181	4
Q46FV4_METBF		Mbar_A0254	F420-dependent N5,N10-methylene-tetrahydromethanopterin reductase.	118	34570	3
Q46FH3_METBF		Mbar_A0386	A1AO H+ ATPase subunit A.	91	63600	2
P07964_METBF		Mbar_A0894	Methyl-coenzyme M reductase subunit gamma (EC 2.8.4.1) (Coenzyme-B sulfoethylthiotransferase gamma) MCRG_METBF.	89	27663	3
Q46EK0_METBF		Mbar_A0714	Aconitate hydratase.	71	102838	2
**Band 4**						
P0C0W7_METBF		Mbar_A1502	Trimethylamine methyltransferase mttB (EC 2.1.1.-) (TMA methyltransferase).	1171	53625	34
Q46AI4_METBF		Mbar_A2180	Glutamate-ammonia ligase.	829	57085	17
Q46FD0_METBF		Mbar_A0199, Mbar_A0429	Carbon-monoxide dehydrogenase, subunit gamma.	592	51200	11
Q46G06_METBF	Peptide 340-349 (FIQSDGGWNR) was unique to Q46G06_METBF. It differentiated from Q46FC7_METBF	Mbar_A202	Carbon-monoxide dehydrogenase, subunit beta.	561	52614	13
Q464Z4_METBF		Mbar_A3685	Protein translation elongation factor 1A.	462	46128	11
P07955_METBF		Mbar_A0897	Methyl-coenzyme M reductase subunit beta (EC 2.8.4.1) (Coenzyme-B sulfoethylthiotransferase beta).	427	45261	8
Q46DX8_METBF		Mbar_A0942	2-isopropylmalate synthase.	363	54208	7
Q46E72_METBF,		Mbar_A0843 or	Monomethylamine methyltransferase mtmB1 (EC 2.1.1.-)	256	49945	7

ID number (UniProt)	Comment	ID number (NCBI)	Protein Description	Mascot Prot Score	MW (Da)	Peptides
Q1DGD7_METBF, Q46E73_METBF, P0C0W3_METBF		Mbar_A0846	(MMA methyltransferase 1) (MMAMT 1).. MTMB1_METBF=Q46E72			
P0C0W5_METBF		Mbar_A1506	Dimethylamine methyltransferase mtbB1 (EC 2.1.1.-) (DMA methyltransferase 1) (DMAMT 1).	241	50169	7
P07964_METBF		Mbar_A0894	Methyl-coenzyme M reductase subunit gamma (EC 2.8.4.1) (Coenzyme-B sulfoethylthiotransferase gamma) MCRG_METBF.	237	27663	5
Q46BP1_METBF		Mbar_A1759	Hypothetical protein.	236	26745	4
Q46FY1_METBF		Mbar_A0227	Glutamate synthase (NADPH).	230	50623	7
Q46EW5_METBF		Mbar_A0597	Phosphomethylpyrimidine synthase 1 thiC1 AAZ69577	162	46586	3
Q468E2_METBF		Mbar_A2850	Enolase (EC 4.2.1.11) (2-phospho-D-glycerate hydro-lyase).	139	46410	3
Q46FH4_METBF		Mbar_A0385	H(+)-transporting ATP synthase, subunit B.	134	50308	2
Q466W7_METBF		Mbar_A3191	Cobyric acid synthase.	128	53269	3
Q46CK2_METBF		Mbar_A1431	D-3-phosphoglycerate dehydrogenase.	118	55761	3
Q466X0_METBF		Mbar_A3188	Putative thymidine phosphorylase TYPH_METBF AAZ72072	112	54594	3
P07962_METBF		Mbar_A0893	Methyl-coenzyme M reductase subunit alpha (EC 2.8.4.1) (Coenzyme-B sulfoethylthiotransferase alpha).	103	61791	2
Q46DL0_METBF		Mbar_A1064	Methanol:corrinoid methyltransferase.	83	50303	2
**Band 5**						
P07955_METBF		Mbar_A0897	Methyl-coenzyme M reductase subunit beta (EC 2.8.4.1) (Coenzyme-B sulfoethylthiotransferase beta).	911	45261	15
Q464Z4_METBF		Mbar_A3685	Protein translation elongation factor 1A.	875	46128	19
P0C0W5_METBF		Mbar_A1506	Dimethylamine methyltransferase mtbB1 (EC 2.1.1.-) (DMA methyltransferase 1) (DMAMT 1).	762	50169	21
Q465E0_METBF		Mbar_A3638	Methanol-5-hydroxybenzimidazolylcobamide co-methyltransferase, isozyme 3.	751	50247	15
Q468E2_METBF		Mbar_A2850	Enolase (EC 4.2.1.11) (2-phospho-D-glycerate hydro-lyase).	740	46410	14
Q46FH4_METBF		Mbar_A0385	H(+)-transporting ATP synthase, subunit B.	598	50308	9
Q46E72_METBF, Q1DGD7_METBF, Q46E73_METBF, P0C0W3_METBF		Mbar_A0843 or Mbar_A0846	Monomethylamine methyltransferase mtmB1 (EC 2.1.1.-) (MMA methyltransferase 1) (MMAMT 1).. MTMB1_METBF=Q46E72	585	49945	14
Q46EW5_METBF		Mbar_A0597	Phosphomethylpyrimidine synthase 1 thiC1 AAZ69577	582	46586	11
Q46FD0_METBF		Mbar_A0199, Mbar_A0429	Carbon-monoxide dehydrogenase, subunit gamma.	566	51200	11

ID number (UniProt)	Comment	ID number (NCBI)	Protein Description	Mascot Prot Score	MW (Da)	Peptides
P0C0W7_METBF		Mbar_A1502	Trimethylamine methyltransferase mttB (EC 2.1.1.-) (TMA methyltransferase).	461	53625	9
Q46FF6_METBF		Mbar_A0403	Adenylosuccinate synthetase purA AAZ69386	310	46646	8
Q46DL0_METBF		Mbar_A1064	Methanol:corrinoid methyltransferase.	271	50303	7
Q46CY7_METBF		Mbar_A1292	Molybdenum formylmethanofuran dehydrogenase subunit.	265	47519	6
Q468C0_METBF		Mbar_A2872	Coenzyme F390 synthetase/phenylacetyl-CoA ligase.	198	48998	4
P07964_METBF		Mbar_A0894	Methyl-coenzyme M reductase subunit gamma (EC 2.8.4.1) (Coenzyme-B sulfoethylthiotransferase gamma) MCRG_METBF.	170	27663	4
Q46BD3_METBF or Q46BD4_METBF		Mbar_A1869 or Mbar_A1868	Iron(III) dicitrate-binding protein.	166	43383	4
Q464Y8_METBF		Mbar_A3691	DNA-directed RNA polymerase, subunit A.	158	46698	3
Q46CD5_METBF		Mbar_A1503	Dimethylamine corrinoid protein.	129	23068	2
Q465P3_METBF		Mbar_A3530	PmbA/TldD family protein.	117	46101	3
Q46FC9_METBF or Q46GO8_METBF		Mbar_A0430 or Mbar_A0200	CO dehydrogenase/acetyl-COA synthase delta subunit.	109	47267	3
Q466Y5_METBF		Mbar_A3173	Aspartyl-tRNA synthetase aspS AAZ72057	107	50764	2
**Band 6**						
Q46FY8_METBF		Mbar_A0220	Ketol-acid reductoisomerase; ILVC_METBF AAZ69204	496	36971	12
Q465J3_METBF		Mbar_A3581	Putative pyridoxine biosynthesis protein.	458	31895	10
Q46DI1_METBF		Mbar_A1093	Phosphoserine phosphatase.	404	32860	9
Q48941_METBA		Mbar_A1288	molybdenum formylmethanofuran dehydrogenase chain F; S62197- fmdF	388	37226	10
Q46BM3_METBF		Mbar_A1778	Phosphate-binding protein.	388	31499	8
Q46FV4_METBF		Mbar_A0254	F420-dependent N5,N10-methylene-tetrahydromethanopterin reductase.	353	34570	9
P94919_METBF		Mbar_A2233	N(5),N(10)-methenyltetrahydromethanopterin cyclohydrolase, MCH_METBF, AAZ71158,	336	34881	7
Q46BI0_METBF		Mbar_A1821	Phosphate acetyltransferase.	334	35326	5
O93716_METBF		Mbar_A1255	MTRH_METBF, AAZ70218, Tetrahydromethanopterin S-methyltransferase subunit H, Methanosarcina barkeri (strain Fusaro / DSM 804)	324	34234	8
Q48949_METBA		Mbar_A1054	mtaA methylcobalamin-coenzyme M methyltransferase isozyme I s62368	317	35849	6
P07964_METBF		Mbar_A0894	MCRG Methyl-coenzyme M reductase subunit gamma (EC 2.8.4.1) (Coenzyme-B sulfoethylthiotransferase gamma).	305	27663	7

**ID number (UniProt)**	**Comment**	**ID number (NCBI)**	**Protein Description**	**Mascot Prot Score**	**MW (Da)**	**Peptides**
Q46CL6_METBF		Mbar_A1417	Dimethylallyltranstransferase / geranyltranstransferase.	299	35366	5
Q46D91_METBF		Mbar_A1183	GTP cyclohydrolase mptA Methanosarcina barkeri (strain Fusaro / DSM 804).	251	35716	5
Q46E75_METBF		Mbar_A0841	Methylcobamide:CoM methyltransferase mtbA (EC 2.1.1.-) (Methylcobamide:CoM methyltransferase II isozyme A) (MT2-A).. MTBA_METBA; S62369, O30640, Q48928	245	36510	5
Q48943_METBF		Mbar_A1290	formylmethanofuran dehydrogenase (EC 1.2.99.5) (molybdenum) chain C ; s62199; FMDC_METBF	209	31531	4
Q465R0_METBF		Mbar_A3512	Ornithine carbamoyltransferase.	193	33792	5
Q46BF4_METBF		Mbar_A1847	F420-nonreducing hydrogenase I.	174	41354	4
P55301_METBF		Mbar_A0980	H4MPT formyltransferase AAZ69952	159	31677	4
Q466U0_METBF		Mbar_A3220	Phage shock protein A.	151	28109	4
Q46F30_METBF		Mbar_A0531	Phosphoribosylaminoimidazole-succinocarboxamide synthase.	122	27157	3
Q46D96_METBF		Mbar_A1178	GMP synthase (Glutamine-hydrolyzing).	117	34072	3
Q465Y2_METBF		Mbar_A3437	Hypothetical protein.	101	35933	2
Q46GC8_METBF		Mbar_A0077	30S Ribosomal protein S4P AAZ69064	99	24348	2
Q46DH9_METBF		Mbar_A1095	Methylenetetrahydromethanopterin dehydrogenase.	95	29802	2
Q46ET9_METBF		Mbar_A0623	Isocitrate/isopropylmalate dehydrogenase family protein.	94	37134	2
Q464Z4_METBF		Mbar_A3685	Protein translation elongation factor 1A.	94	46128	3
Q46G31_METBF		Mbar_A0176	Fructose-bisphosphate aldolase.	74	33668	2
Q46D53_METBF		Mbar_A1222	Hypothetical protein.	71	34314	2
Q46E72_METBF, Q1DGD7_METBF, Q46E73_METBF, P0C0W3_METBF		Mbar_A0843 or Mbar_A0846	Monomethylamine methyltransferase mtmB1 (EC 2.1.1.-) (MMA methyltransferase 1) (MMAMT 1).. MTMB1_METBF=Q46E72	60	49965	2

Table S3: Mbar_A1758 peptides detected from analyses of multiple gel bands.

Peptides	Sequence
25-31	A.ADSVEIR.G
75-94	K.DVSGNSGNVIGEGGIVYSTK.I
136-147	K.LVIDSDDKITLR.T
148-163	R.TGETLDIGQGYTLQAK.Q
164-171	K.QVDVDGEK.V
164-171	K.pyro-QVDVDGEK.V
164-178	K.QVDVDGEKVWLEFDR.D
172-178	K.VWLEFDR.D
277-289	R.DSTNELAEGLSFK.V
290-299	K.VADTSSNVLR.F
305-315	K.EFTDPGTYEVR.G
420-435	R.TGETFDLGEGYSIQAK.Q
444-450	K.VWLEFDK.D
488-495	K.VHVNQVFR.G
539-549	K.ISNEDTFTLTR.D
550-562	R.DSDEDIGEGMYFK.V
550-562	R.DSDEDIGEGMoxYFK.V
563-572	K.VADTPTSELR.Y
573-579	R.YYPAIER.I

Table S4: Microarray transcript abundance of all cell surface and cell surface-related genes annotated in the *M. acetivorans* genome.

Name[a]	Annotated Function[a]	Proteomic data[b]	Average count[c]		Transmembrane domains[d]
			Methanol	Acetate	
MA0336	cell surface protein	Y	1866.2	155.2	2
MA0488	cell surface protein	N	152.1	1.1	0
MA0509	surface antigen gene	N	NA	NA	1
MA0512	surface antigen gene	N	NA	NA	2
MA0588	cell surface protein	Y	413.1	339.4	1
MA0850	cell surface protein	N	417.4	130.2	1
MA0851	cell surface protein	N	313.3	70	1
MA1286	cell surface glycoprotein (S-layer protein)	N	75.9	26.6	3
MA1292	cell surface protein	N	744.9	191.3	1
MA1293	cell surface protein	N	161.7	32.4	0
MA1420	cell surface protein	Y	497.7	338	1
MA1454	cell surface lipoprotein	Y	441.2	44.1	1
MA1508	cell surface protein	N	237.1	145.8	1
MA1517	cell surface protein	N	447.9	162.8	0
MA1590	surface antigen gene	N	414.7	116.4	1
MA1591	surface antigen gene	N	NA	NA	1
MA1717	cell surface protein	N	612.8	155.2	2
MA1730	cell surface protein	N	157.8	137.5	1
MA1738	cell surface protein	N	650	347.1	1
MA1749	cell surface glycoprotein	N	31.9	3.9	1
MA1751	cell surface glycoprotein	N	618.4	473.1	1
MA1753	cell surface protein	N	350.4	508.3	1
MA1754	cell surface protein	N	389.7	906.3	0
MA1756	cell surface protein	N	558.2	1219.6	0
MA1761	surface antigen gene	Y	NA	NA	1
MA1762	cell surface protein	Y	NA	NA	1
MA1764	cell surface protein	Y	534.7	1959.7	1
MA1766	cell surface protein	Y	94.7	1748.6	2
MA1767	cell surface protein	N	NA	NA	1
MA1838	cell surface protein	N	281.5	135	1
MA1961	cell surface glycoprotein (S-layer protein)	N	49.5	12.9	1
MA2017	cell surface protein	N	178	154.2	0
MA2019	pneumococcal surface protein	Y	338.9	215.9	0
MA2284	cell surface protein	N	242.1	174.9	1
MA2335	cell surface protein	N	269.7	80.1	0
MA2457	cell surface glycoprotein (S-layer protein)	N	45.6	23.2	1
MA2507	cell surface protein	N	66.1	51.7	0
MA2706	cell surface protein	N	253.5	51.8	1
MA2707	cell surface protein	N	NA	NA	0
MA2793	cell surface protein	N	385.7	235.6	1
MA2885	cell surface protein	N	531.4	212.8	1
MA2886	cell surface protein	N	1073.8	673.4	0

Name[a]	Annotated Function[a]	Proteomic data[b]	Average count[c]		Transmembrane domains[d]
			Methanol	Acetate	
MA2916	cell surface protein	N	13	14.6	2
MA2926	cell surface protein	N	275.1	48.4	1
MA2979	cell surface protein	N	222.1	121.2	3
MA3101	cell surface protein	N	204.6	135.2	2
MA3118	surface antigen gene	Y	NA	NA	1
MA3120	surface antigen gene	N	NA	NA	1
MA3122	surface antigen gene	N	NA	NA	1
MA3133	cell surface protein	Y	745	884.7	2
MA3229	cell surface protein	N	30.2	-18.6	4
MA3430	cell surface protein	N	173.9	76.3	1
MA3664	cell surface protein	N	2206.5	404.5	2
MA3698	cell surface protein	N	123	19.5	0
MA3699	cell surface protein	N	561.2	315.1	1
MA3700	cell surface protein	N	1369.2	490	1
MA3703	cell surface glycoprotein	N	766.7	185.4	1
MA3782	cell surface protein	N	383	126.6	2
MA3842	cell surface protein	Y	130.9	43.5	2
MA4033	surface antigen gene	N	616.5	556	1
MA4222	cell surface protein	N	322.7	203.3	1
MA4284	cell surface protein	N	NA	NA	0
MA4285	cell surface protein	N	181.7	115.8	0
MA4287	cell surface protein	N	2538.2	839.6	1
MA4289	cell surface protein	N	297	144	0
MA4291	cell surface protein	N	341.8	509.9	1
MA4292	cell surface protein	N	567	502.7	0
MA4297	cell surface protein	N	306.6	169.7	0
MA4299	cell surface protein	N	NA	NA	1
MA4301	cell surface protein	N	858.7	369	0
MA4305	cell surface protein	N	741.8	140.2	1
MA4309	cell surface protein	N	409.2	282.2	1
MA4312	cell surface protein	N	89.3	21.5	1
MA4315	cell surface protein	N	1227.9	668.6	1
MA4363	cell surface protein	N	267.4	47.2	1
MA4478	cell surface protein	N	3091	610.1	0
MA4481	cell surface protein	N	481.7	52	1
MA4577	cell surface protein	N	276.9	97.8	2
MA4588	cell surface protein	N	264.9	82.6	1
MA4589	cell surface protein	N	514.2	258.6	1

[a]Gene annotations are from the original genome annotation file [2] [b]Data from [17]. [c]Microarray data [17] from (see Methods and Materials). [d]Predicted trans-membrane domain (TM) from the genome annotation using TMHMM [18]. N, not detected; Y, detected; NA, data not articulated.
